# Suppressors of Cytokine Signaling in Sickness and in Health of Pancreatic β-Cells

**DOI:** 10.3389/fimmu.2016.00169

**Published:** 2016-05-09

**Authors:** Cheng Ye, John P. Driver

**Affiliations:** ^1^Department of Animal Sciences, University of Florida, Gainesville, FL, USA

**Keywords:** suppressors of cytokine signaling, β-cells, SOCS expression, insulin signaling, inflammatory cytokines, diabetes, β-cell growth

## Abstract

Suppressors of cytokine signaling (SOCS) are a family of eight proteins that negatively regulate Janus kinase and signal transducers and activators of transcription signaling in cells that utilize this pathway to respond to extracellular stimuli. SOCS are best known for attenuating cytokine signaling in the immune system. However, they are also expressed in many other cell types, including pancreatic β-cells, where there is considerable interest in harnessing SOCS molecules to prevent cytokine-mediated apoptosis during diabetes and allogeneic transplantation. Apart from their potential as therapeutic targets, SOCS molecules play a central role for regulating important functions in β-cells, including growth, glucose sensing, and insulin secretion. This review will discuss SOCS proteins as central regulators for diverse cellular processes important for normal β-cell function as well as their protective anti-apoptotic effects during β-cell stress.

## Introduction

β-cells within the islets of Langerhans secrete the hormone insulin, not only in response to glucose but also in response to other nutrients, hormones, and neuronal stimuli (1). The primary role of β-cells is to maintain plasma glucose levels within tight physiological ranges for optimal functioning of the body’s cells. Normal physiology places high demands on β-cells compared to non-secretory cells, which may contribute to pathological conditions associated with pancreatic islet destruction and dysfunction, including types 1 and 2 diabetes. Type 1 diabetes develops from the autoimmune destruction of β-cells, while type 2 diabetes results from insulin resistance that ultimately causes β-cell exhaustion. Although the etiology of each disease is different, both disorders involve β-cell dysregulation through cytokine-induced inflammation (2, 3).

The capacity of β-cells to respond to their environment and dynamically adjusts blood glucose levels depend on multiple receptors and biological signaling pathways that interactively mediate glucose sensing and insulin secretion. Many of these pathways initiate signal transduction by activating the Janus kinase and signal transducers and activators of transcription (JAK–STAT) pathway (4). It is now established that JAK–STAT signaling is inhibited by a family of intracellular proteins collectively known as suppressors of cytokine signaling (SOCS) that reduce the magnitude and/or duration of signals induced by diverse receptors (5–8). Although originally known for inhibiting cytokine signaling in immune cells through a classical negative feedback loop, SOCS proteins emerged as important modulators of additional pathways, including those that control insulin secretion and β-cell development and proliferation. Eight members of the SOCS family have been described, including SOCS-1–7 and CIS. SOCS molecules have in common that they contain a central Src-homology 2 (SH2) domain, a conserved COOH-terminal SOCS box, and a variable N-terminal domain (9, 10). Two SOCS molecules, SOCS-1 and SOCS-3, contain a kinase inhibitory region (KIR) (11, 12). SOCS proteins inactivate JAK–STAT signaling by binding directly to the tyrosine-phosphorylated residues on JAKs *via* the SH2 domain that blocks access of STATs to receptor-binding sites. They also suppress signaling by directly inhibiting JAK kinase activity and by targeting receptors and JAKs for degradation by the proteasome [reviewed in Ref. (13, 14)]. Here, we focus on what is known about the expression of SOCS proteins in β-cells and how SOCS molecules regulate β-cell function under normal and pathophysiological conditions.

## SOCS Expression in β-Cells

In general, *Socs* genes are expressed at low or undetectable levels in resting cells but become rapidly induced after stimulation with cytokines or hormones. Their transcription is upregulated by the STAT and NFκB-transcription factors, and the resultant SOCS proteins generated subsequently suppresses the same pathway that stimulated their production. Table 1 describes what is currently known about the expression of different SOCS family members in β-cells. In primary human and mouse β-cells, SOCS-1, -2, and CIS are expressed at low baseline levels, although SOCS-3 message and protein are virtually undetectable in unmanipulated healthy islets. Interestingly, expression of SOCS-1, -2, and -3 proteins is upregulated in islet cells from human type 1 diabetes (T1D) patients compared to healthy controls (15). Also, islets purified from NOD mice that develop spontaneous T1D express increased levels of SOCS during the progression of pancreatic insulitis, including CIS and SOCS-2 transcripts from 7 weeks of age and SOCS-1 transcripts from 10 weeks of age (16). These findings suggest that β-cells synthesize SOCS proteins in response to the pro-inflammatory environment that accompanies β-cell autoimmunity.

**Table 1:** **Expression of SOCS family members in β-cells**.

SOCS family members	β-cell source	Stimulation conditions	Product measured	Expression	Reference
SOCS-1	Human islet cells	Baseline; 10 healthy subjects	mRNA	Constitutively expressed at low levels	(15)
		IFNγ + IL-1β + TNFα	mRNA	Markedly increased compared to baseline	
		Baseline; 10 healthy subjects	Protein	Detected at low levels	
		Baseline; 3 T1D patients	Protein	High compared to healthy controls	
	Mouse islet cells	Baseline (NOD.SCID)	mRNA	Constitutively expressed at low levels	(16)
		IL-1β (NOD.SCID)	mRNA	None detected	
		TNFα (NOD.SCID)	mRNA	None detected	
		IFNγ (NOD.SCID)	mRNA	Peak at 4 h post incubation	
		Baseline (NOD)	mRNA	Detected by 70 days of age	
	NIT-1 mouse β-cell line	IL-1β	mRNA	Does not induce unless combined with IFNγ	(16)
		TNFα	mRNA	Does not induce unless combined with IFNγ	
		IFNα	mRNA	Peak at 2 h post incubation	
		IFNγ	mRNA	Peak at 4 h post incubation and remains elevated	
		IFNγ	Protein	Increased after 4 h and remains elevated beyond 24 h	

SOCS-2	Human islet cells	Baseline; 10 healthy subjects	mRNA	Constitutively expressed at low levels	(15, 56)
		IFNγ + IL-1β + TNFα	mRNA	Markedly increased compared to baseline	
		Baseline; 10 healthy subjects	Protein	Detected at low levels	
		Baseline; 3 T1D patients	Protein	High compared to healthy controls	
	Mouse islet cells	Baseline (NOD.SCID)	mRNA	Low	(16, 57)
		Baseline (C57BL/6J)	Protein	Very low	
		IL-1β (NOD.SCID)	mRNA	Peak at 4 h post incubation	
		TNFα (NOD.SCID)	mRNA	Peak at 1 h post incubation	
		IFNγ (NOD.SCID)	mRNA	Peak at 1 h post incubation	
		Baseline (NOD)	mRNA	Detected by 50 days of age	
		Pregnancy (C57BL/6J, CD-1)	mRNA	Increased by day 14.5 of pregnancy	(24)
	NIT-1 mouse β-cell line	IL-1β	mRNA	Peak at 1 h post incubation	(16)
		TNFα	mRNA	Peak at 1 h post incubation	
		IFNα	mRNA	Not changed compared to baseline	
		IFNγ	mRNA	Peak expression at 1 h post incubation	

SOCS-3	Human islet cells	Baseline; 10 healthy subjects	mRNA	Constitutively expressed at low levels	(15)
		IFNγ + IL-1β + TNFα	mRNA	Sixfold increase at 4–6 h post incubation, remains elevated beyond 24 h	
		IL-1	mRNA	Fourfold increase at 4 h post incubation, decays to baseline levels within 24 h	(15, 19)
		Baseline; 10 healthy subjects	Protein	Detected at low levels	
		Baseline; 3 T1D patients	Protein	High compared to healthy controls	(15)
		Leptin	mRNA	Strong induction after 12 h	(20)
	Rat islet cells	Baseline	mRNA	Low	(19, 21, 28)
		IFNγ	mRNA	Not different compared to baseline	(19)
		IFNγ	mRNA	Markedly increased compared to baseline	(21, 28)
		IL-1β + IFNγ	mRNA	20-fold increase at 4 h post incubation and decays to baseline levels within 24 h	(19)
		IL-1β + IFNγ	Protein	Below baseline	(22)
	RIN rat β-cell line	IL-1β	mRNA	4.3-fold increase at 2 h post incubation	(17, 18, 20)
		IL-1β + IFNγ	Protein	Below baseline	(22)
	INS-1 rat cell line	Leptin	mRNA	Increased by 30 min post incubation	(20)
	Mouse islet cells	Leptin (*ob/ob*)	mRNA	Peak at 6 h post incubation (*in vivo* treatment)	(20)
		Resistin (ICR)	Protein	Increased expression by 12 h	(26)

CIS	Mouse islet cells	Baseline (NOD.SCID)	mRNA	Constitutively expressed	(16)
		IL-1β (NOD.SCID)	mRNA	Increased within 1 h post incubation	
		TNFα (NOD.SCID)	mRNA	Increased within 1 h post incubation	
		IFNγ (NOD.SCID)	mRNA	Increased within 1 h post incubation	
		Baseline (NOD)	mRNA	Detected at day 50 days of age	
		Pregnancy (C57BL/6J, CD-1)	mRNA	Increased by day 14.5 of pregnancy	(24)
	NIT-1 mouse β-cell line	IL-1β	mRNA	Peak at 1 h post incubation	(16)
		TNFα	mRNA	Peak at 1 h post incubation	
		IFNα	mRNA	Not different compared to baseline	
		IFNγ	mRNA	Peak at 1 h post incubation	

Several studies have investigated which specific cytokines induce SOCS expression in β-cells. These have revealed that some cytokines induce the expression of several *Socs* genes, while others induce only one or a few. Chong et al. demonstrated that interferon γ (IFNγ) induces prolonged SOCS-1 mRNA expression (>48 h) in NIT-1 cells, a NOD mouse-derived insulinoma cell line, which peaks 4 h after cells are cultured with the cytokine. They also found that IFNα stimulates NIT-1 cells to transiently express SOCS-1 that peaks 2 h after stimulation and then rapidly decays (16). Primary mouse islets separately treated with IFNγ, but not IL-1β or TNFα, upregulated SOCS-1 expression. In the same study, SOCS-2 and CIS expression were rapidly induced in NIT-1 cells and mouse islets, incubated separately with IFNγ, IL-1β, or TNFα. However, IFNα did not increase CIS and SOCS-2 transcripts above baseline levels (16).

Interleukin-1β rapidly stimulated SOCS-3 transcription in the RINm5F rat β-cell line that spiked 2 h after incubation (17, 18). SOCS-3 mRNA is also induced in primary human β-cells exposed to IL-1β, although the effect on SOCS-3 expression was greater when IL-1β was combined with IFNγ and TNFα (19, 20) In rat islets, IL-1β stimulated a 20-fold increase in SOCS-3 mRNA after 4 h of culture that returned to baseline levels within 24 h (19, 21). IFNγ also upregulated SOCS-3 transcription; however, the increase in expression was transient compared to IL-1β and the effect disappeared within 24 h after stimulation. The combination of IL-1β and IFNγ additively increased SOCS-3 mRNA levels in rat islets. By contrast, Lv et al. found that combined IL-1β and IFNγ treatment actually downregulated SOCS-3 protein expression in the RIN rat β-cell line and in primary rat islets after 1 and 24 h of incubation, respectively (22).

Suppressors of cytokine signaling proteins are also expressed in response to hormones that alter energy metabolism to accommodate different physiological conditions. Pregnancy induced high levels of CIS and SOCS-2 transcription in mice when β-cell proliferation was stimulated by lactogens (23, 24). SOCS-3 transcription is also induced when rat and human β-cells are treated with leptin, a satiety hormone (19, 20, 25). Another study showed that resistin, an adipokine that contributes to insulin resistance, induces SOCS-3 protein expression in primary mouse β-cells (26). It is important to note that multiple post-transcriptional mechanisms are used to regulate the levels of some SOCS proteins (27). Therefore, measuring mRNA transcription alone may not adequately describe SOCS expression in β-cells.

## Effects of SOCS on Insulin Production and Signaling

Suppressors of cytokine signaling proteins appear to “fine-tune” insulin synthesis and secretion by β-cells in response to a variety of external stimuli. Some of these are hormones that stimulate or suppress insulin production for regulating normal energy metabolism, while others are cytokines that alter insulin signaling during inflammatory conditions. Although changes in SOCS expression undeniably affects insulin signaling in β-cells, disagreements between the model systems used to investigate this phenomenon has made it difficult to firmly establish the natural contribution of SOCS to pancreatic islet function. Some of these discrepancies are highlighted below.

Suppressors of cytokine signaling regulation of GH-stimulated insulin secretion was explored by RØnn and colleagues (28) because it was known that GH stimulates insulin production by β-cells (29, 30) and because SOCS molecules inhibit GH signaling in other tissues (31–33). It was found that SOCS-3 inhibits GH-induced insulin mRNA transcription in a RIN-5AH β-cell line that was transfected with an inducible SOCS-3 expression system. SOCS-3 overexpression abolished STAT3 and STAT5 activation and DNA-binding ability. The JAK2–STAT5 pathway is the main signaling pathway triggered by GH in β-cells where STAT5 molecules are known to translocate to STAT5-specific elements of the insulin promoter (29, 34–36). Thus, the authors postulated that SOCS-3 blocks GH-induced insulin production by reducing the amount of activated STAT5 available for stimulating insulin expression. These conclusions contrast with a more recent study by the same authors, which found that female mice with β-cell-specific overexpression of SOCS-3 actually exhibit enhanced glucose tolerance compared to littermate controls, in spite of developing smaller islets (37). It was postulated that SOCS-3 mediates inhibition of several different signaling pathways in β-cells besides GH, some of which could enhance glucose-stimulated insulin secretion.

Suppressors of cytokine signaling-3 is important for how leptin affects insulin production by β-cells. Leptin is a hormone synthesized by white adipose tissue that regulates body fat mass by controlling appetite and energy expenditure through effects on the hypothalamus [reviewed in Ref. (38)]. Findings that leptin-deficient *ob/ob* mice and leptin receptor-defective *db/db* mice develop hyperinsulinelima before the onset of a type 2 diabetes phenotype led to discoveries that leptin also inhibits preproinsulin gene expression by β-cells (39–41). SOCS-3 has been established as a mediator of central leptin resistance (25, 42). Laubner et al. found that SOCS-3 is also important for leptin-mediated repression of insulin production in β-cells, the purpose of which is probably to adjust glucose homeostasis to the amount of body fat (20). Using the rat insulinoma cell line INS-1, it was demonstrated that leptin signaling in β-cells stimulates leptin receptor associated JAK2 tyrosine kinase activity that phosphorylates STAT3 and STAT5b, which bind to the SOCS-3 promoter inducing transcription. SOCS-3 in turn inhibits JAK–STAT signaling that is required for insulin expression. Dysregulation of SOCS-3 signaling may contribute to the development of type 2 diabetes in obese individuals whose β-cells become resistant to leptin signaling, leading to chronic insulin hypersecretion that eventually causes β-cell failure.

Suppressors of cytokine signaling molecules appear to protect against the impaired insulin secretion by β-cells exposed to pro-inflammatory cytokines. Previous studies have shown that IFNγ reduces glucose-stimulated insulin secretion in β-cell lines as well as rodent and human islets (43–45). Cottet et al. assessed whether overexpressing SOCS-1 in the insulin-secreting cell line, βTc-Tet, could inhibit the IFNγ-driven JAK–STAT signal transduction pathway and prevent IFNγ-induced reductions in insulin gene expression and secretion (46). Constitutive SOCS-1 expression blocked phosphorylation and nuclear translocation of STAT-1 compared to non-transduced βTc-Tet cells, and IFNγ-induced reductions in insulin mRNA levels and glucose-stimulated insulin secretion were prevented. These findings differed from a later study that showed no effects of SOCS-1 overexpression on cytokine-induced inhibition of glucose-stimulated insulin secretion using primary islets from mice transgenically overexpressing SOCS-1 that were incubated with different concentrations of glucose and a mixture of IL-1β, TNFα, and IFNγ (47). Possible reasons for this discrepancy are that the studies used different sources of β-cells, IFNγ concentrations, culture conditions, and the origin and expression levels of SOCS-1 were also different.

Apart from regulating insulin expression, SOCS molecules may play a role in the desensitization of β-cells to autocrine/paracrine insulin signaling that follows prolonged glucose exposure. The chronic hyperglycemia and inflammatory cytokines that accompany type 2 diabetes are known to cause β-cells to become resistant to insulin signaling (48–51), which is usually required for normal β-cell functioning and survival (52, 53). The involvement of SOCS in this phenomenon has been investigated because SOCS molecules cause insulin resistance in other insulin-sensitive tissues that are exposed to inflammatory cytokines (54). A link between SOCS-1 and glucose-attenuated insulin signaling was demonstrated by Venieratos et al. who showed that prolonged exposure of the β-cell line βTc-6 to high glucose concentrations inhibited insulin-induced tyrosine phosphorylation of the insulin receptor (IR), IR substrate-2 (IRS-2), as well as PI3-kinase activation (55). These impairments were associated with enhanced endogenous interleukin-1β (IL-1β) by βTC-6 cells that, in turn, stimulated expression of SOCS-1. SOCS-1 was critical for desensitization of β-cells to insulin signaling because specific ablation of this molecule by small interfering RNA restored insulin signaling suppressed by high glucose. Although these authors found no effect of high glucose on SOCS-3 expression, another study demonstrated that SOCS-3 had very similar effects to SOCS-1 for inhibiting insulin signaling in RINm5F cells, a rat pancreatic β-cell line, after exposure to IL-1β (17).

The role of SOCS family members besides SOCS-1 and SOCS-3 for regulating insulin production and signaling in β-cells remains unclear. Conditional β-cells ablation of CIS showed no non-redundant functions for this gene (24). Another study investigated the importance of SOCS-2 and found no difference in glucose-induced insulin secretion in islets isolated from SOCS-2 knockout (KO) mice compared to wild-type controls (56). SOCS-2-KO mice were also normal for insulin and glucose tolerance. However, a separate report showed that transgenic mice constitutively expressing SOCS-2 in β-cells develop severe defects in glucose metabolism that were attributed to profoundly altered insulin secretion to various secretagogues, perturbed Ca flux in response to glucose, and impaired proinsulin maturation (57). While these results could indicate a role for SOCS-2 in β-cell function, it is possible that the defects observed were artifacts related to the transgenic overexpression of SOCS-2 protein off the insulin promoter, which has caused similar disruptions in a variety of pancreas-specific gene manipulations (58, 59).

## SOCS Regulation of Cytokine Signaling and β-Cell Apoptosis

Suppressors of cytokine signaling proteins play a central role for regulating how β-cells respond to cytokines. Pro-inflammatory cytokines, such as IFNγ, IL-1β, and TNFα, are secreted by lymphocytes and macrophages within pancreatic infiltrates during T1D development and after allogeneic islet transplantation and contribute to β-cell destruction by inducing a variety of pro-apoptotic processes (60, 61). SOCS-1 and SOCS-3 buffer β-cells against these effects by downregulating pro-inflammatory cytokine-signaling pathways. Besides understanding the natural effects of SOCS proteins in β-cells, there exists considerable interest in enhancing expression of these molecules to mitigate cytokine effects during T1D and after islet transplantation. Most of what is known about how SOCS proteins modulate β-cell cytokine responses has been discovered by a small number of researchers. A summary of their findings is described below.

### Suppressors of Cytokine Signaling-1

In the early 2000s, studies were conducted to determine whether SOCS-1 was involved in protecting β-cells against the pro-apoptotic effects of IFNγ. These were motivated by reports showing that IFNγ induces SOCS-1 that negatively regulated JAK/STAT signaling in a wide variety of cell types (62, 63). Chong et al. showed that SOCS-1 overexpression in NIT-1 cells inhibited IFNγ signaling, which blocked STAT-1 activation and IFNγ-induced apoptosis (16). However, natural expression of SOCS-1 did not affect the kinetics and intensity of IFNγ-signaling in primary β-cells, suggesting that SOCS-1 might not be involved in regulating inflammatory cytokines under physiological conditions. However, it was subsequently found that β-cells from SOCS-1-KO mice were more susceptible to cell death when exposed to IFNγ in combination with TNFα compared to islets from wild-type mice (64). TNFα does not activate the JAK–STAT pathway. Thus, increased TNFα + IFNγ-induced cell death in SOCS-1 (KO) islets may have been the result of hypersensitivity to TNFα-stimulated iNOS expression and nitric oxide (NO) production *via* dysregulation of the p38 mitogen-activated protein kinase pathway. TNFα alone did not induce iNOS expression or cell death, indicating that the pathways stimulated by IFNγ are still necessary for iNOS induction in SOCS-1-KO β-cells. Similar results were obtained by Cottet et al. who showed that SOCS-1 overexpression prevents iNOS expression and apoptosis in βTc-Tet cells exposed to IFNγ in combination with TNFα and IL-1β (46).

More recent studies have employed SOCS-1 transgenic mice to examine how SOCS-1 overexpression affects β-cell resistance against spontaneous T1D and allogeneic transplantation. Flodström-Tullberg et al. found that spontaneous T1D was reduced in SOCS-1 transgenic NOD mice and that protection was associated with cytokine-induced STAT-1 phosphorylation within β-cells (65). Chong et al. obtained similar results showing that rat insulin promoter-driven SOCS-1 expression in β-cells prevents progression to diabetes in NOD mice and CD8^+^ TCR transgenic NOD.NY8.3 mice (66). Another study demonstrated that SOCS-1 transgenic mice are protected from virally induced CD8^+^ T cell-mediated T1D (67). In transplantation studies, islets from C57BL6 (B6) SOCS-1 transgenic mice survived longer than wild-type islets when engrafted into allogeneic BALB/c recipient mice (68). The same SOCS-1 transgenic islets did not survive better than controls when transplanted into clinically diabetic NOD mice, probably because of already high circulating levels of autoreactive T cells. Another report found that SOCS-1 overexpression induced by an adenovirus system increased the survival of rat islets transplanted into allogeneic recipients with streptozotocin-induced diabetes (69).

Follow-up studies have identified several mechanisms through which SOCS-1 overexpression may improve β-cell survival in diabetes and islet transplantation models. Chong et al. showed that SOCS-1 inhibits IFNγ and TNFα-induced Fas and IL-15 expression by β-cells (66). In this way, SOCS-1 may prevent CD8^+^ T cells from killing β-cells through Fas–Fas ligand interactions and reduce IL-15-mediated homing and activation of diabetogenic T cells within the pancreas. Another group found that SOCS-1 transgenic islets express less of the IFNγ inducible chemokine *Cxcl10* that is usually produced by islets during diabetes development and may be involved in recruitment of autoreactive T cells to the inflamed pancreas (70). Using islets from SOCS-1 transgenic mice on the B6 background, Zaitseva et al. demonstrated that SOCS-1 may block cytokine-mediated islet apoptosis by decreasing IFNγ, TNFα, IL-1β-induced caspase-3, -8, and -9 expression in β-cells (47). Usually, caspase-3 activation, which can be initiated by caspases 8 and 9, is essential for the induction of apoptosis in β-cells (71, 72). Unlike previous reports, these authors found no evidence that SOCS-1 prevents cytokine-mediated NO production and suggested that changes in NO production might not contribute to the protective effect of SOCS-1 overexpression in β-cells. In a separate report, Solomon et al. showed that transgenic expression of SOCS-1 rendered islets resistant to IFNγ and TNFα-induced cell death and that resistance was correlated with a significant inhibition of the transcription factor interferon regulatory factor-1 (IRF-1) (73). They proposed a model in which SOCS-1 prevents cytokines from inducing IRF-1 that normally inhibits anti-apoptotic proteins, such as Bcl-2 and Bcl-xL, by blocking the cytoprotective NFκB pathway (74–76). Several studies have found that SOCS-1 reduces β-cell expression of class I MHC molecules following exposure to pro-inflammatory cytokines (16, 66, 67, 73). IFNγ produced during allograft rejection and T1D development is known to upregulate class I MHC antigen on β-cells, which makes them more vulnerable to lysis by CD8^+^ T cells. SOCS-1 overexpression was demonstrated to reduce class I MHC expression by pancreatic islets incubated with IFNγ and TNFα and after LCMV infection (67). One report showed that physiological levels of SOCS-1 protein are sufficient to reduce class I MHC expression following cytokine exposure, because SOCS-1-deficient β-cells were more sensitive to TNFα-induced class I MHC expression compared to wild-type islets (64).

### Suppressors of Cytokine Signaling-3

Different research groups using similar model systems published reports characterizing SOCS-1 and SOCS-3 regulation of β-cell cytokine signaling, concurrently. An early study by Karlsen et al. used INS-1 cells with doxycycline (DOX)-inducible SOCS-3 expression to show that the apoptotic effects of IL-1β and IFNγ were, respectively, fully and partially blocked by SOCS-3 overexpression at moderate and high concentrations of both cytokines (21). The protective effects of SOCS-3 overexpression were correlated with decreased IL-1β-induced iNOS promoter activity and NO production. In primary islet cells from rats and humans, SOCS-3 mRNA expression was induced by exposure to IL-1β alone and in combination with IFNγ and TNFα, indicating that SOCS-3 might contribute to the natural resistance of islets against the toxic effects of pro-inflammatory cytokines (19, 21).

A number of mechanisms have been identified, in addition to reduced NO production, through which SOCS-3 overexpression may protect islets from cytokine-mediated apoptosis. By comparing global gene expression following IL-1β exposure of INS-1 cells with and without DOX-induced SOCS-3 expression, it was found that multiple IL-1β-induced NFκB-dependent early apoptotic and immune genes were inhibited, including ICAM, complement C3, Mob-1, MIP-1, CX3C, NFκB–p105, IRF-1, and fibrinogen-γ (77). In a separate study, DOX-inducible SOCS-3 INS-1 cells and primary rat islets transduced with a SOCS-3-encoding adenovirus showed that IL-1β-induced expression of Fas and the chemokines Mcp-1, Mip-2, and St-38, which depend on the NFκB pathway, become inhibited by SOCS-3 overexpression (78). Collectively, these results suggest that by blocking NFκB activity, SOCS-3 may protect β-cells from attracting, engaging, and activating autoreactive T cells.

Suppressors of cytokine signaling-3 overexpression partially protects β-cells from immune responses that develop after allogeneic transplantation. RØnn et al. showed that islets from B6 mice with β-cell-specific SOCS-3 expression survived longer compared to wild-type islets when transplanted into BALB/c mice (19). However, the same transgenic islets did not survive better than wild-type islets when transplanted into spontaneously diabetic NOD mice. Outcomes of these transplantation studies are remarkably similar to experiments using SOCS-1 transgenic islets (68, 69) and demonstrate that SOCS-3 expression can also protect islets against allogeneic MHC responses but cannot circumvent the autoimmune responses that develop in the NOD mouse. It is not yet known whether SOCS-3 transgenic expression impacts spontaneous diabetes. However, one report examined whether β-cell-specific SOCS-3 expression could protect B6 mice from multiple low-dose streptozotocin-induced diabetes development (79). Surprisingly, SOCS-3 transgenic mice tended to develop accelerated disease compared to wild-type controls. To explain their findings, the authors proposed that increased SOCS-3 expression might, under some conditions, lead to a failed upregulation of protective pathways that would normally become activated after β-cell exposure to inflammatory cytokines. In support of this hypothesis, the investigators showed that SOCS-3 overexpression in mouse and rat islets reduced IL-1β-induced expression of IL-1Rα, a glycolipid that antagonizes IL-1β-induced β-cell damage (80). Another explanation for the increased sensitivity of SOCS-3 transgenic mice to MLDSTZ-induced diabetes is that SOCS-3 overexpression could obstruct normal β-cell functioning, for instance, by interfering with JAK/STAT-dependent insulin signaling.

Although much emphasis has been placed on understanding SOCS-3 modulation of IL-1β and IFNγ signaling in β-cells, Bruun et al. demonstrated that SOCS-3 expression also inhibits TNFα signaling (81). They further showed that TNFα transiently induces the expression of SOCS-3 mRNA in primary rat β-cells, through stimulating NFκB and MAP kinases. Apoptosis mediated by TNFα alone or in combination with IL-1β was suppressed by overexpression of SOCS-3 in INS-1 cells by repressing TNFα-induced IκB degradation, NFκB DNA binding, and NFκB-transcription of MnSOD, a classical example of an NFκB responsive gene.

Overexpression studies have established that SOCS-3 is capable of regulating signaling induced by at least three major cytokines involved in the pathogenesis of T1D. Consequently, SOCS-3 represents a promising target for therapeutic interventions to protect β-cell mass. However, unlike SOCS-1, there is little known about how SOCS-3 contributes to the natural resistance of β-cells against cytokine-mediated toxicity, in part, because data are lacking about SOCS-3-deficient β-cells. SOCS-2 is also expressed in β-cells, but the role of this SOCS member on β-cell apoptosis has yet to be firmly established. Puff et al. reported that IL-1β-mediated cell death *in vitro* was unchanged after siRNA mediated SOCS-2 knockdown in INS-1E cells. Also, destruction of β-cells after MLDSTZ injections was not altered in SOCS-2-KO mice (56). In contrast, Alkharusi et al. showed that 6-month-old, but not 2-month-old, SOCS-2-KO mice were less sensitive to the effects of MLDSTZ treatment (82).

## SOCS Proteins Modulate β-Cell Growth and Replication

Growth hormone and prolactin (PRL) are hormones that stimulate β-cell replication through JAK–STAT-signaling receptors that are subject to suppression by SOCS proteins (83–88). Both GH and PRL stimulate β-cell growth and proliferation *in vitro* (84, 89, 90). Furthermore, mice deficient for PRL receptor (PRLR) develop smaller β-cells and reduced pancreatic insulin content compared to wild-type controls (91). β-cell-specific GH receptor knockout (GHRKO) mice develop normal β-cell mass when raised on a standard chow diet (30). However, they exhibit a dramatic defect in β-cell hyperplasia when fed a high-fat diet (HFD), indicating that GH may be dispensable for normal β-cell development but required for β-cell compensatory growth in response to HFD challenge.

Pancreatic islets modulate expression of receptors for GH and PRL during periods of β-cell expansion, such as during development, pregnancy, and lactation (87, 88, 92). It has been reported that SOCS proteins inhibit GH- and PRL-mediated signal transduction and proliferation in multiple tissue types (31, 33, 93), which prompted studies to investigate whether SOCS molecules also suppress GH- and PRL-mediated growth in β-cells. RØnn et al. investigated the role of SOCS signaling in the β-cells lines RIN-5AH and INS-1 with inducible SOCS-3 expression (28). In a dose-dependent way, SOCS-3 inhibited GH-induced DNA binding of both STAT3 and STAT5 in RIN-5AH cells stably transfected with a ponasterone-inducible SOCS-3 expression system. 5-bromodeoxyuridine (BrdU) incorporation was used to show that SOCS-3 inhibits GH-induced proliferation of INS-1 cells in a dose-dependent manner with DOX-inducible expression of SOCS-3. Lindberg et al. examined the *in vivo* effects of specific transgenic overexpression of SOCS-3 in β-cells (37). In spite of large variation in SOCS-3 transgene expression, β-cell volume of transgenic mice was reduced by approximately 30% compared with β-cell volume in wild-type female mice. There was no difference in β-cell volume between male and female mice in either group or between male transgenic mice and male wild-type littermates. This may have been because β-cell volume in wild-type female mice is usually relatively large compared to male mice, and, therefore, SOCS-3 overexpression may have had a more pronounced effect on female than on male mice. SOCS-3 overexpression had no effect on the amount of proliferating β-cells, probably because inhibition of GH and/or PRL happened at an earlier stage of development than when the mice were analyzed (2 months of age) (88). Transduction of primary neonatal rat islet cultures with recombinant adenoviruses expressing various SOCS proteins, followed by stimulation with GH, demonstrated that SOCS-3 inhibits islet proliferation by GH (37). The effect appeared to be specific for SOCS-3, as SOCS-1, SOCS-2, or CIS expression did not affect GH-induced β-cell proliferation. Additional studies using SOCS-2-deficient (56) and SOCS-2 overexpressing mice (57) appear to rule out that SOCS-2 is involved in β-cell hypertrophy or replication.

Hormones besides GH and PRL induce β-cell growth. Some of these, such as placental lactogen (83, 87, 94) and glucagon-like peptide-1 (95), signal independently of the JAK/STAT pathway and are not subject to inhibition by SOCS molecules. SOCS proteins may regulate IR signaling that employs the JAK/STAT pathway and which appears to be important for maintaining adult β-cell mass (96) and inducing compensatory β-cell proliferation during HFD challenge (97). This question remains to be answered by future studies.

## Conclusion

**Figure 1:**
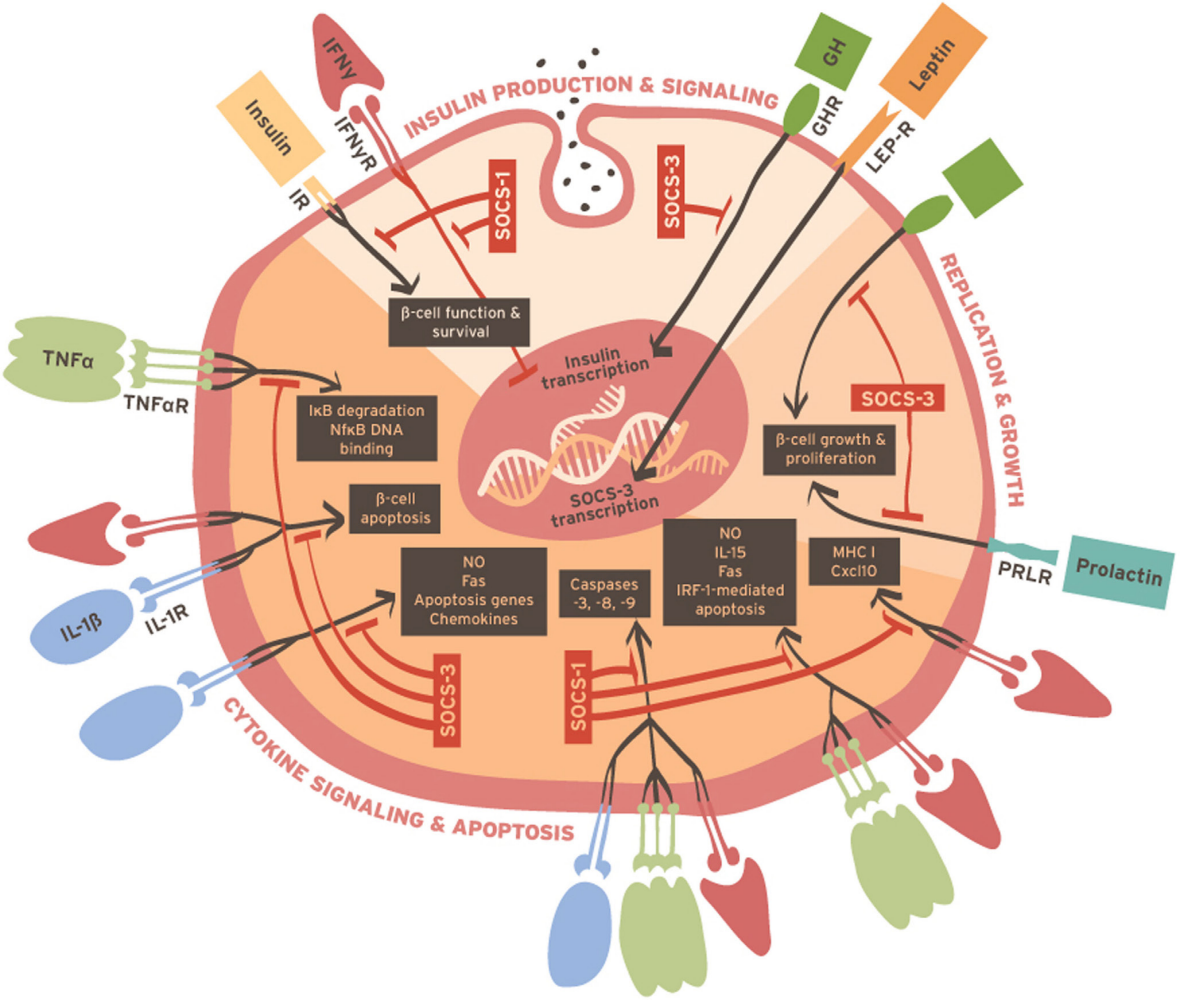
**Summary of cellular processes involved in β-cell function, survival, and replication that are reportedly regulated by SOCS molecules**.

The discovery that SOCS proteins are expressed in β-cells and interactively regulate a diverse range of signaling pathways has greatly advanced our understanding about how β-cell growth, function, and survival are controlled. They also provide insight about how β-cells manage to simultaneously regulate often contradictory signaling pathways for different families of receptors, i.e., IR, GH/PRL receptors, leptin receptors, and cytokine receptors. Figure 1 describes some of these pathways. Although considerable progress has been achieved, gaps remain in our understanding about the natural role of SOCS in β-cells, which is partly due to a reliance on genetically modified mice or cell lines that express non-physiological levels of SOCS proteins compared to primary β-cells. Nevertheless, important information has been gleaned from diverse model systems about the possibility of therapeutically harnessing SOCS proteins to improve the function and survival of β-cells *in vivo* and for propagating and differentiating β-cells *in vitro* for transplantation. These discoveries represent important advances toward better treatments for diabetes.

## Author Contributions

CY and JD co-wrote and edited the manuscript.

## Conflict of Interest Statement

The authors declare that the research was conducted in the absence of any commercial or financial relationships that could be construed as a potential conflict of interest.
